# Nicotinamide N-Methyltransferase in Cardiovascular Diseases: Metabolic Regulator and Emerging Therapeutic Target

**DOI:** 10.3390/biom15091281

**Published:** 2025-09-04

**Authors:** Jawaria, Yusra Zarlashat, Márton Philippovich, Edit Dósa

**Affiliations:** 1 Health Biotechnology Division, National Institute for Biotechnology and Genetic Engineering-C (NIBGE), Faisalabad 38000, Pakistan; jawariasiddiqui100@gmail.com; 2 Pakistan Institute of Engineering and Applied Sciences (PIEAS), Islamabad 45650, Pakistan; 3 Department of Biochemistry, Government College University Faisalabad, Faisalabad 38000, Pakistan; 4 Heart and Vascular Center, Semmelweis University, 1122 Budapest, Hungary

**Keywords:** cardiovascular disease, atherosclerosis, nicotinamide N-methyltransferase, NAD^+^ metabolism, oxidative stress, sirtuins, NNMT inhibitors

## Abstract

Cardiovascular disease (CVD) remains a leading cause of morbidity and mortality worldwide, arising from complex interactions among metabolic, genetic, and environmental factors. Nicotinamide N-methyltransferase (NNMT) has recently emerged as a key metabolic regulator in CVD pathogenesis. By consuming nicotinamide and methyl groups, NNMT perturbs epigenetic, metabolic, and redox pathways that are critical for cardiovascular health. NNMT-mediated NAD^+^ depletion impairs mitochondrial function, sirtuin (SIRT) activity, redox balance, and energy metabolism, thereby creating a pro-atherogenic environment. NNMT and its product 1-methylnicotinamide (1-MNA) show a complex duality: they modulate SIRT activity—particularly SIRT1 and SIRT3—to influence gluconeogenesis, cholesterol synthesis, lipogenesis, and mitochondrial antioxidant defenses. NNMT upregulation also elevates homocysteine levels, activating pro-inflammatory and pro-oxidative cascades (e.g., TLR4–NF-κB and STAT3–IL-1β). Growing evidence links NNMT to major CVD risk factors, including hyperlipidemia, hypertension, diabetes mellitus, and obesity. Thus, NNMT has a multifaceted role in cardiovascular health: while its enzymatic activity is often pathogenic (via NAD^+^/SAM consumption and homocysteine production), its metabolite 1-MNA can exert protective effects (via NRF2 activation and anti-thrombotic mechanisms). This duality highlights the need to delineate the molecular processes that balance these opposing actions. Experimental studies using small-molecule NNMT inhibitors and RNA interference have shown promising cardiometabolic benefits in preclinical models, including improved insulin sensitivity, reduced atherosclerosis, and attenuated cardiac dysfunction. However, no clinical trials have yet targeted NNMT specifically in CVD. Future research should clarify the tissue-specific functions of NNMT and translate these insights into novel therapeutic strategies.

## 1. Introduction

Cardiovascular disease (CVD) encompasses a broad group of disorders affecting the heart and blood vessels, including arteries, veins, and capillaries. It remains the leading cause of death worldwide, accounting for more than 20.5 million deaths each year, with this figure projected to rise to 35.6 million by 2050 [1]. The high global burden of CVD highlights the urgent need to identify novel therapeutic targets to improve disease outcomes.

Nicotinamide N-methyltransferase (NNMT) has recently emerged as a key regulator of cardiovascular health due to both its enzymatic activity and the biological effects of its metabolic byproducts [2,3,4]. Elevated NNMT expression is not merely a biomarker but a functional contributor to CVD pathogenesis. Tissue-specific upregulation in the endothelium, vascular smooth muscle cells, and cardiomyocytes correlates with disease severity in conditions such as heart failure and atherosclerosis [5]. NNMT is a cytosolic enzyme that uses S-adenosyl-L-methionine (SAM) to methylate nicotinamide (NAM; vitamin B3), producing 1-methylnicotinamide (1-MNA) and S-adenosylhomocysteine (SAH) [6]. By consuming SAM, NNMT limits the availability of methyl groups required for histone and DNA methylation, thereby altering gene expression patterns relevant to metabolic and inflammatory pathways [7]. At the same time, nicotinamide methylation reduces the pool of precursors available for nicotinamide adenine dinucleotide (NAD^+^) biosynthesis, potentially lowering intracellular NAD^+^ levels. Because NAD^+^ is essential for energy metabolism, mitochondrial function, and redox balance, its depletion can severely impair cardiomyocyte energetics [2]. Such NAD^+^ loss disrupts oxidative phosphorylation, compromises contractile function, and ultimately promotes cardiac dysfunction [8]. In addition, SAH generated by NNMT is a precursor of homocysteine (Hcy), whose elevated levels are strongly associated with CVD development [9].

Genetic studies further support a pathogenic role of NNMT. Single nucleotide variants (SNVs) in the *NNMT* gene have been linked to metabolic disturbances and increased risk of obesity [10], type 2 diabetes mellitus [11], hyperlipidemia [12], and hypertension [13]—all well-established CVD risk factors. Elevated NNMT expression in the liver and adipose tissue also connects the enzyme to systemic metabolic dysfunction [2]. Moreover, cellular stress, such as impaired autophagy, can upregulate NNMT expression, accelerating nicotinamide clearance and disrupting NAD^+^ homeostasis. In experimental models, NNMT inhibition restores NAD^+^ levels and improves mitochondrial and cardiac function [14]. Taken together, these findings identify NNMT as a central mediator of mitochondrial and cardiac dysfunction through its impact on NAD^+^ metabolism. Recent genetic association studies further implicate *NNMT* haplotypes as independent risk factors for coronary artery disease (CAD), underscoring their potential as novel determinants of CVD susceptibility. This review summarizes the metabolic mechanisms and signaling pathways linking NNMT to cardiometabolic disease, highlights its therapeutic potential, and provides an overview of recent advances in NNMT-related cardiovascular research.

## 2. Metabolic Link Between NNMT and CVDs

NNMT-mediated methylation of nicotinamide influences both NAD^+^-dependent signaling pathways and Hcy metabolism. This reaction produces 1-MNA and SAH, thereby reducing the availability of nicotinamide for NAD^+^ biosynthesis and lowering intracellular NAD^+^ levels. Nicotinamide is a critical precursor for NAD^+^, which acts as a coenzyme in essential metabolic processes, including glycolysis, the citric acid cycle, and oxidative phosphorylation [15]. The balance between nicotinamide methylation and its salvage into NAD^+^ suggests that NNMT restricts fuel oxidation and promotes lipid accumulation by suppressing NAD^+^-dependent processes [16]. As a result, cells increasingly rely on fatty acid oxidation. When excessive, this reliance contributes to lipotoxicity and mitochondrial stress, both of which are key drivers of cardiovascular dysfunction [17]. Supporting this, the *NNMT* genetic variant rs1941404 has been associated with altered resting energy expenditure in humans [12]. Consistently, Kraus et al. [18] showed that siRNA-mediated *Nnmt* knockdown or pharmacological inhibition of NNMT increased oxygen consumption. Enhanced energy expenditure in white adipose tissue and liver was directly linked to uncoupling protein 1 (UCP1)-independent thermogenesis in adipocytes, revealing a novel pathway through which NNMT inhibition promotes negative energy balance [2].

**Figure 1:**
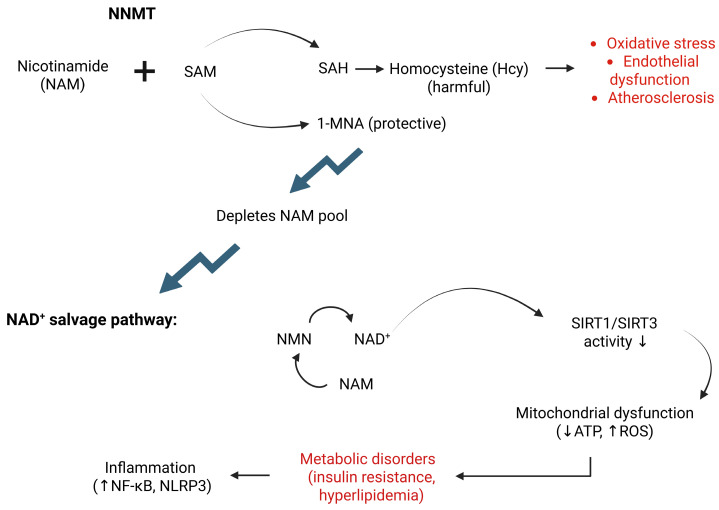
NNMT metabolic pathways and cardiovascular implications. Schematic overview of NNMT enzymatic activity and its downstream effects on NAD^+^ biosynthesis and Hcy metabolism. The figure highlights key pathways through which NNMT contributes to metabolic dysregulation and cardiovascular dysfunction. ATP—adenosine triphosphate; Hcy—homocysteine; 1-MNA—1-methylnicotinamide; NAD^+^—nicotinamide adenine dinucleotide; NAM—nicotinamide; NF-κB—nuclear factor kappa-light-chain-enhancer of activated B cells; NLRP3—NOD-, LRR-, and pyrin domain-containing protein 3; NMN—nicotinamide mononucleotide; NNMT—nicotinamide N-methyltransferase; ROS—reactive oxygen species; SAH—S-adenosylhomocysteine; SAM—S-adenosylmethionine; SIRT—sirtuin.

NNMT also regulates plasma Hcy levels. SAH, the byproduct of NNMT activity, is hydrolyzed to Hcy by SAH hydrolase. Elevated Hcy concentrations impair antioxidant defenses in cardiomyocytes by inhibiting glutathione peroxidase (GPx), a major antioxidant enzyme, which leads to the accumulation of lipid peroxides and reactive oxygen species (ROS) [15,19]. These toxic species damage endothelial cells and promote inflammation [20]. High Hcy levels also activate pro-inflammatory signaling pathways and induce endothelial dysfunction, establishing Hcy as an independent risk factor for CVD [21]. Metabolic regulation by NNMT extends to glucose homeostasis. In human adipose tissue, NNMT expression correlates positively with insulin resistance and type 2 diabetes mellitus [22]. Conversely, NNMT inhibition improves glucose tolerance and reduces body weight in high-fat diet-fed mouse models [23]. Mechanistically, NNMT knockdown suppresses hepatic gluconeogenesis by downregulating key genes such as *Pck1* (phosphoenolpyruvate carboxykinase 1) and *G6pc* (glucose-6-phosphatase catalytic subunit), leading to reduced glucose output in primary hepatocytes [24]. This effect is mediated by activation of the AMP-activated protein kinase (AMPK) pathway, triggered by restoration of the cellular NAD^+^/NADH ratio. As a consequence, systemic insulin sensitivity improves, alleviating metabolic stress on the cardiovascular system (Figure 1).

## 3. Crosstalk Between NNMT and the SIRT Signaling Pathway

NNMT can negatively regulate the cellular NAD^+^ pool, which is essential for the activity of sirtuins (SIRTs). These enzymes play critical roles in glucose metabolism, energy homeostasis, and inflammatory signaling [25]. Hong et al. [24] demonstrated that SIRT1 mediates the regulatory effects of NNMT and its metabolite 1-MNA on glucose metabolism. Specifically, SIRT1 upregulation restored the expression of *Pck1* and *G6pc*, which had been suppressed by NNMT knockdown in hepatocytes [24]. Dysregulated SIRT activity impairs mitochondrial function, leading to reduced ATP production, excess ROS, and cell death—all of which contribute to CVD progression [25].

SIRTs are NAD^+^-dependent deacetylases that couple lysine deacetylation with NAD^+^ hydrolysis. In this reaction, the acetyl group from acetylated lysine is transferred to ADP-ribose, generating O-acetyl-ADP-ribose and releasing free nicotinamide. Their activity therefore depends directly on NAD^+^ availability [26], which is influenced by both NNMT activity and cellular nutrient status [15,26]. Mammals express seven isoforms (SIRT1–SIRT7), sharing a conserved catalytic core but differing in terminal domains, which confer distinct functions. Collectively, SIRTs act as metabolic sensors that regulate glucose homeostasis, insulin sensitivity, and oxidative stress. Among them, SIRT1 has particular importance in CVD due to its role in vascular homeostasis and regulation of signaling pathways essential for vascular function [27]. SIRT1 also governs gluconeogenesis by deacetylating transcriptional regulators such as CREB-regulated transcription coactivator 2 (CRTC2), forkhead box O1 (FOXO1), and peroxisome proliferator-activated receptor gamma coactivator 1-alpha (PGC-1α) [24]. These transcriptional programs control glucose metabolism, mitochondrial activity, and overall energy balance [28,29]. In addition, SIRT1 inhibits sterol regulatory element-binding proteins (SREBP-1 and SREBP-2), central regulators of lipid metabolism, thereby suppressing lipogenesis and cholesterol synthesis [24].

Emerging evidence indicates that NNMT regulates gluconeogenesis, cholesterol metabolism, and lipogenesis partly through stabilization of the SIRT1 protein, potentially mediated by 1-MNA. Interestingly, 1-MNA acts not only as a metabolite but also as a feedback inhibitor of NNMT by binding to its active site. Both NNMT and 1-MNA promote upregulation of SIRT1 protein expression [24]. Functionally, SIRT1 overexpression protects against atheroma formation in apolipoprotein E (ApoE)-knockout mouse models. Conversely, deletion of *Sirt1* in ApoE-knockout mice increases susceptibility to atherosclerosis, confirming its protective role. In line with these findings, systemic SIRT1 levels are reduced in patients with CAD [25] (Figure 1).

## 4. NNMT in Oxidative Stress and Inflammation

Oxidative stress, defined as an imbalance between ROS production and antioxidant defenses, promotes tissue damage and triggers inflammatory cascades that drive CVD pathogenesis [30]. NNMT is involved in multiple oxidative stress and inflammatory pathways, although its precise role remains incompletely defined. Its major metabolite, 1-MNA, activates nuclear factor erythroid 2-related factor 2 (NRF2) and inhibits nuclear factor kappa-light-chain-enhancer of activated B cells (NF-κB) signaling, thereby protecting cells against oxidative damage and inflammation. NRF2 induces antioxidant genes such as *Sod* (superoxide dismutase), *Hmox1* (heme oxygenase-1), *Cat* (catalase), and *Nqo1* (NAD[P]H quinone dehydrogenase 1). In cardiomyocytes exposed to palmitic acid, 1-MNA administration significantly upregulated NRF2 target proteins (NQO1, HO-1, and glutamate-cysteine ligase catalytic subunit [GCLC]), confirming its cytoprotective role [31].

NNMT also appears to support endothelial defense. Inhibition of NNMT reduced nuclear SIRT1 levels and increased its phosphorylated form (pSIRT1), suggesting that the NNMT–SIRT1 axis protects vascular endothelium under oxidative stress [32]. In contrast, NNMT overexpression can exacerbate oxidative stress. By depleting NAD^+^, it inhibits SIRT3, a mitochondrial deacetylase that activates antioxidant enzymes such as SOD2 and isocitrate dehydrogenase 2 (IDH2) [33]. This inhibition leads to mitochondrial hyperacetylation, impairs electron transport chain function, and increases superoxide (O_2_^−^) leakage.

Elevated NNMT expression is closely associated with hyperhomocysteinemia (HHcy), since SAH is converted into Hcy [34]. High Hcy activates Toll-like receptor 4 (TLR4), promoting vascular inflammation and mitochondrial dysfunction. TLR4 activation further induces pro-inflammatory cytokines, including interleukin-6 (IL-6), tumor necrosis factor-alpha (TNF-α), and interleukin-1 beta (IL-1β) [35]. Hcy also stimulates C-reactive protein (CRP) production in vascular smooth muscle cells through the N-methyl-D-aspartate (NMDA) receptor–ROS–extracellular signal-regulated kinase 1/2 (ERK1/2)–p38–NF-κB signaling pathway [36]. Elevated CRP levels aggravate atherosclerosis. Clinical studies support this mechanism: plasma Hcy levels positively correlate with high-sensitivity CRP in female cardiovascular patients [37].

Experimental models of inflammatory injury also show NNMT upregulation. Its activity increases markedly in concanavalin A-induced liver injury [38] and monocrotaline (MCT)-induced pulmonary hypertension [39]. In vitro, stimulation with transforming growth factor-beta (TGF-β), TNF-α, or IL-6 enhances NNMT expression in human skeletal muscle myoblasts [40]. These findings suggest that NNMT overexpression may act as a compensatory response to inflammation and tissue injury [41,42].

Beyond NAD^+^ depletion, NNMT overexpression fosters a pro-oxidative environment via interconnected pathways. It enhances ROS production, activates the signal transducer and activator of transcription 3 (STAT3)–IL-1β–prostaglandin E_2_ (PGE_2_) cascade, and stimulates secretion of collagens, extracellular matrix proteins, and pro-inflammatory cytokines [43]. Paradoxically, 1-MNA can exert protective effects by upregulating NRF2-dependent antioxidant enzymes (HO-1, NQO1) and suppressing NF-κB-mediated oxidative stress in high-fat diet-fed mice [31]. This duality is further complicated by evidence that impaired autophagy activates NF-κB through sequestosome 1 (SQSTM1/p62) accumulation, which upregulates NNMT, creating a feedback loop that depletes NAD^+^ and intensifies oxidative stress [14]. Taken together, these findings suggest that NNMT acts not only as a downstream effector of oxidative stress but also as a central regulator that links metabolic pathways with redox homeostasis (Figure 1).

## 5. Role of NNMT in CVDs

### 5.1. NNMT and Hyperlipidemia

Hyperlipidemia is characterized by abnormally high plasma lipid levels—including cholesterol, triglycerides (TG), and low-density lipoprotein (LDL)—all of which are major risk factors for CVD [44]. Experimental studies show that NNMT knockdown reduces TG and free fatty acid levels in the liver and adipose tissue, underscoring its key role in fat metabolism [18]. Genetic evidence also supports this link: the rs1941404 single-nucleotide polymorphism (SNP) in the *NNMT* gene is significantly associated with hyperlipidemia in the Chinese Han population, likely through its effect on resting energy expenditure [12].

NNMT contributes to hyperlipidemia not only through lipid metabolism but also via Hcy regulation. Elevated Hcy levels are strongly associated with dyslipidemia [45,46]. For example, in rat models, Hcy administration (50 mg/kg/day) induced hyperlipidemia [47]. In vitro studies using alpha mouse liver (AML12) hepatocyte cells further confirmed this mechanism: NNMT knockdown increased SAM and SAH levels, reduced 1-MNA production, and downregulated *Srebf1* (sterol regulatory element-binding protein 1 [SREBP1]), a key lipogenic gene. These changes decreased neutral lipid accumulation and improved lipid profiles [48].

**Figure 2:**
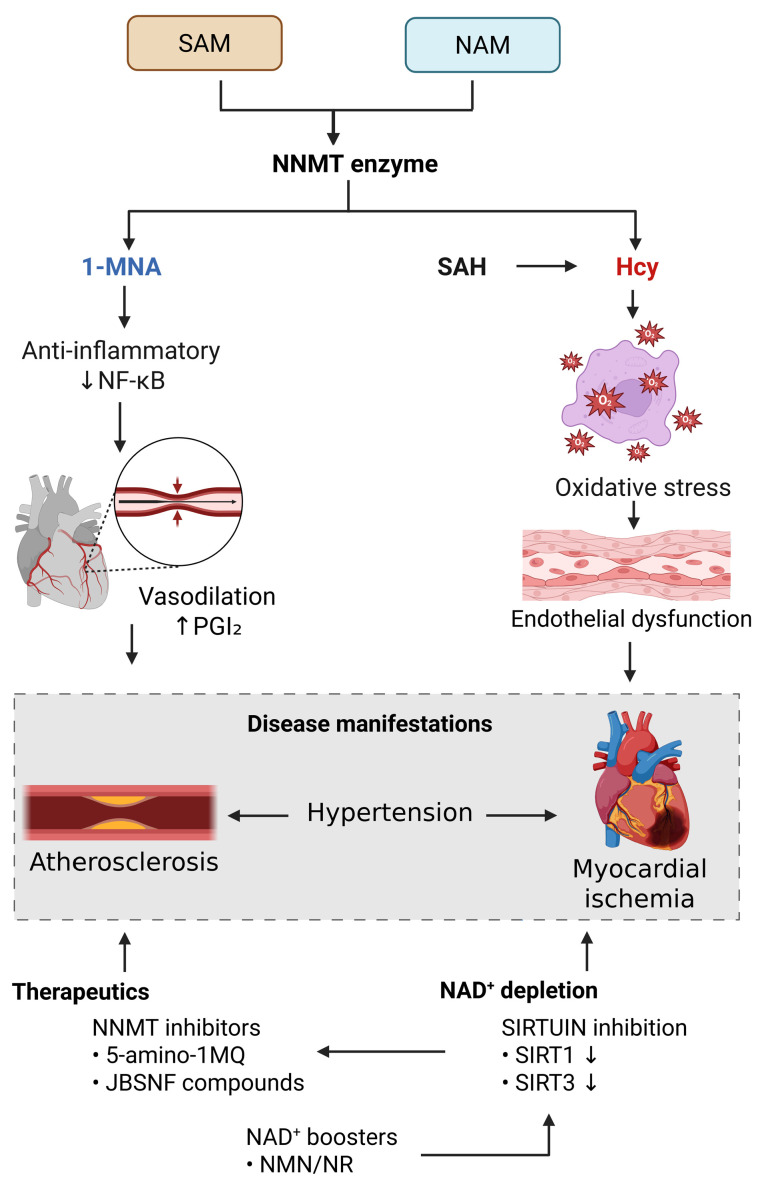
NNMT in CVD pathogenesis and therapy. Overview of NNMT enzymatic activity in cardiovascular pathophysiology, showing conversion of NAM and SAM into 1-MNA and SAH/Hcy and the resulting disease mechanisms. Potential therapeutic strategies are highlighted, including NNMT inhibition and NAD^+^-boosting approaches, with relevance to atherosclerosis, hypertension, and myocardial ischemia. 5-amino-1MQ—5-amino-1-methylquinolinium; Hcy—homocysteine; 1-MNA—1-methylnicotinamide; NAD^+^—nicotinamide adenine dinucleotide; NAM—nicotinamide; NF-κB—nuclear factor kappa-light-chain-enhancer of activated B cells; NMN—nicotinamide mononucleotide; NNMT—nicotinamide N-methyltransferase; NR—nicotinamide riboside; PGI_2_—prostaglandin I_2_; SAH—S-adenosylhomocysteine; SAM—S-adenosylmethionine; SIRT—sirtuin.

Dysregulated lipid metabolism is a hallmark of hyperlipidemia, and NNMT activity strongly influences transcriptional regulators. In particular, NNMT modulates peroxisome proliferator-activated receptor gamma (PPARγ), a central transcription factor that controls lipid biosynthesis, lipoprotein metabolism, energy balance, and adipogenesis [49]. Xu et al. [49] (2022) investigated NNMT function in adipocytes and reported two key findings: (1) NNMT knockdown reduced lipid accumulation and TG content, accompanied by decreased expression of adipogenic transcription factors (PPARγ, CCAAT/enhancer-binding protein alpha [C/EBPα], SREBP1) and lipid metabolism genes—*Fabp4* (fatty acid-binding protein 4 [FABP4]), *Fasn* (fatty acid synthase [FASN]), *Slc27a1* (FATP1, SLC27A1); (2) NNMT overexpression promoted lipid storage while suppressing secretion of adipokines such as *Adipoq* (adiponectin) and *Lep* (leptin). In addition, NNMT silencing altered autophagy pathways. Knockdown decreased expression of autophagy markers (*Becn1* [Beclin1], *Atg7* [autophagy related 7], *Atg12* [autophagy related 12], *Atg14* [autophagy related 14], *Map1lc3b* [LC3B]) while increasing *Sqstm1* (SQSTM1/p62), indicating impaired autophagic flux [49]. These results highlight the multifaceted role of NNMT in lipid metabolism, adipokine regulation, and autophagy, all of which contribute to the development of hyperlipidemia (Table 1, Figure 2).

### 5.2. NNMT and Atherosclerosis

Atherosclerosis is a progressive inflammatory disease characterized by the accumulation of lipids, immune cells, and cellular debris within the arterial intima, ultimately leading to plaque formation. This process causes arterial stiffening and narrowing, which restricts blood flow to the myocardium [63]. NNMT catalyzes the methylation of nicotinamide to generate 1-MNA, a metabolite with reported anti-thrombotic and anti-inflammatory effects. In ApoE/LDL receptor (LDLR)(−/−) mice, hepatic NNMT activity and circulating 1-MNA levels progressively increased compared with wild-type controls. By two months, hepatic NNMT activity doubled, and by six months it had risen fivefold. This upregulation correlated with advanced plaque development, macrophage infiltration, and elevated metalloproteinase-2 (MMP2)/MMP9 activity, representing a hepatic compensatory response to vascular inflammation [3].

Plaque rupture represents a critical event in atherosclerosis. Exposure of tissue factor to circulating blood initiates coagulation cascades and platelet activation, leading to thrombus formation that may obstruct the arterial lumen and cause acute ischemic events. Plaque vulnerability is further exacerbated by neovascularization: fragile intraplaque microvessels can rupture, causing hemorrhage, oxidative stress, and inflammation, all of which increase the risk of rupture and thrombosis [51,64]. Notably, 1-MNA demonstrates thrombolytic properties in vivo by inhibiting platelet-mediated thrombosis through activation of the cyclooxygenase-2 (COX-2)–prostacyclin (PGI_2_) pathway. This suggests that NNMT-derived 1-MNA may help counterbalance thrombosis and inflammation to preserve vascular health [65]. In contrast, increased NNMT activity can drive pro-inflammatory signaling. Reduced NAD^+^ levels activate the *Stat3* pathway, leading to enhanced STAT3 expression, K685 acetylation, and S727 phosphorylation. This cascade elevates IL-1β and COX-2 expression, resulting in increased PGE_2_ synthesis. Simultaneously, NNMT downregulates *Hpgd*, the gene encoding 15-hydroxyprostaglandin dehydrogenase (15-PGDH), the enzyme responsible for PGE_2_ degradation, causing PGE_2_ accumulation and establishing a pro-inflammatory milieu [43]. These changes contribute to plaque instability, rupture, and vascular inflammation, thereby promoting atherothrombosis [66,67]. Clinical evidence supports these mechanistic insights. Liu et al. [9] observed significantly higher serum 1-MNA levels in patients with CAD, with concentrations positively correlating with disease severity. Elevated 1-MNA was associated with increased systemic inflammation (high-sensitivity CRP) and reduced high-density lipoprotein cholesterol, suggesting that NNMT influences atherosclerosis progression through both metabolic and inflammatory mechanisms [9]. In summary, NNMT upregulation is linked to atherosclerosis progression, vascular inflammation [3], plaque instability, and thrombosis [43,66,67]. These findings identify NNMT as a promising therapeutic target in CAD and related CVDs (Table 1, Figure 2).

### 5.3. NNMT and Hypertension

Hypertension is one of the major risk factors for cardiovascular complications [68]. Fedorowicz et al. [39] investigated the NNMT–1-MNA pathway in pulmonary arterial hypertension (PAH) in both rats and humans. In rat models, NNMT activity and 1-MNA levels were elevated in the plasma, liver, and lungs, correlating with disease progression and increased PGI_2_ production. Similarly, patients with idiopathic PAH exhibited higher plasma 1-MNA levels. These findings suggest that activation of the NNMT–1-MNA pathway may act as a compensatory, vasoprotective response in PAH [39]. In addition, human studies show that serum 1-MNA levels are significantly associated with left ventricular systolic dysfunction and CAD in the Chinese population [9,69].

NNMT activity also influences blood pressure via Hcy regulation. Elevated plasma Hcy levels are positively correlated with hypertension [54]. Patients with HHcy display significantly higher blood pressure compared with those without HHcy. Consistently, rat models confirm that experimentally increased Hcy levels raise blood pressure [53]. Genetic evidence further supports this link: the *NNMT* variant rs1941404 is significantly associated with primary hypertension in the Chinese population, likely due to its effect on Hcy metabolism [13].

Another mechanism by which NNMT contributes to hypertension is through NAD^+^ depletion. NNMT-mediated methylation consumes nicotinamide, a critical precursor of NAD^+^ biosynthesis, thereby lowering cellular NAD^+^ availability [55]. NAD^+^ is essential for blood pressure regulation because it supports vasodilation and reduces oxidative stress. In vascular endothelial cells from porcine coronary arteries and rat thoracic aorta, increased NAD^+^ levels induced concentration-dependent vasorelaxation via adenosine receptor activation [70]. Taken together, these findings highlight NNMT as a central mediator of hypertension, acting through the NNMT–1-MNA pathway, Hcy accumulation, and NAD^+^ depletion. As such, NNMT represents a promising therapeutic target for the prevention and management of hypertension and its associated cardiovascular complications (Table 1, Figure 2).

### 5.4. NNMT and Myocardial Ischemia

Myocardial ischemia occurs when oxygen and nutrient supply to the myocardium is restricted, most often due to blocked or narrowed coronary arteries [71]. Severe ischemia can result in irreversible myocardial damage [72]. Multiple studies indicate that NNMT contributes to ischemia pathogenesis by regulating NAD^+^ metabolism, oxidative stress, energy-related pathways, and inflammatory signaling [2,43,60], all of which play critical roles in disease development and progression [73].

Nicotinamide mononucleotide (NMN) is a direct precursor of NAD^+^ in the salvage pathway [74]. NNMT indirectly reduces NMN availability by depleting nicotinamide, which is normally converted to NMN by nicotinamide phosphoribosyltransferase (NAMPT) during NAD^+^ synthesis [41]. Experimental evidence shows that NNMT knockdown increases NAMPT expression and elevates NAD^+^ levels in adipose tissue [18]. Furthermore, combined NMN and melatonin treatment activates the SIRT3–FOXO1 pathway and protects against ischemia–reperfusion injury in aged Wistar rats. These findings suggest that limiting NNMT-induced nicotinamide depletion (and thereby preserving NMN) may help mitigate ischemia–reperfusion injury [59].

NNMT upregulation also enhances methionine cycle activity, leading to elevated plasma Hcy levels [61]. Increased Hcy activates the ERK1/2 signaling pathway, promotes ROS generation, and triggers cytochrome c efflux, ultimately resulting in mitochondrial dysfunction in acute ischemia–reperfusion models [57]. Overactivation of NNMT further decreases intracellular NAD^+^ levels, which reduces both expression and activity of SIRT3 [62]. SIRT3 is a key mitochondrial deacetylase that activates antioxidant enzymes such as SOD2. Reduced SIRT3 activity increases mitochondrial ROS accumulation, exacerbating oxidative damage and impairing ATP production during ischemia–reperfusion injury [58] (Table 1, Figure 2).

## 6. Therapeutic Potential of NNMT in CVDs

Early studies demonstrated that *Nnmt* knockdown in mice not only enhances cellular energy expenditure—offering protection against diet-induced obesity [18]—but also significantly suppresses gluconeogenesis in primary hepatocytes [24]. *Nnmt* knockdown reduces TG levels and lipid accumulation in 3T3-L1 cells [49], while animal models show improved fasting glucose, glucose tolerance, and insulin sensitivity following NNMT inhibition [18,24,75]. Both adenovirus-mediated *Nnmt* knockdown and pharmacological inhibition downregulate key lipogenic genes and attenuate fat accumulation under endoplasmic reticulum stress [50]. Antisense oligonucleotide-mediated *Nnmt* knockdown has also shown benefits: it lowered serum insulin levels and limited weight gain in high-fat diet-fed mice (particularly in females), while primarily improving insulin sensitivity in males [76]. Clinically, NNMT expression is significantly upregulated in adipose tissue of patients with type 2 diabetes mellitus and insulin resistance, supporting NNMT inhibition as a potential therapeutic strategy for metabolic risk factors associated with CVD [22]. Collectively, these findings underscore the promise of RNAi-based approaches and small-molecule NNMT inhibitors in cardiometabolic disease [23,77,78].

MicroRNAs (miRNAs) offer another therapeutic avenue. These small non-coding RNAs regulate gene expression post-transcriptionally [79]. For example, miR-29b-3p and miR-378g bind to the 3′ untranslated region (3′UTR) of *Nnmt* mRNA, suppressing its expression. Inhibition of these miRNAs increases *Nnmt* expression and promotes osteogenic differentiation of bone marrow mesenchymal stem cells [80,81]. These observations highlight RNA interference targeting *Nnmt* as a potential novel therapy in CVD.

A feedback inhibitor, SAH, is a naturally occurring competitive antagonist of the SAM-binding domain of NNMT [6]. However, it lacks specificity, also targets other methyltransferases, and is rapidly degraded in vivo, limiting its therapeutic value [6,82]. By contrast, other inhibitors show greater promise. Both 1-MNA and 5-amino-1MQ (NNMTi) bind to the nicotinamide-binding site of NNMT and effectively block its enzymatic activity [83,84].

1-MNA also acts as a feedback inhibitor of NNMT, increasing oxygen consumption in adipocytes and raising NAD^+^ levels, thereby promoting gluconeogenesis in hepatocytes [18]. However, its in vivo efficacy remains inconsistent. Some studies reported improved metabolic parameters in high-fat diet-fed mice [24], while others found no significant effects on lipid profiles, fasting glucose, or hemoglobin A1c [85]. These discrepancies likely stem from rapid urinary excretion, poor membrane permeability, and chemical instability [2,86].

In contrast, 5-amino-1MQ (NNMTi) shows robust potential. Neelakantan et al. [86] demonstrated that NNMTi treatment reduces body weight, white adipose tissue mass, total cholesterol, and adipocyte size in obese mice. NNMTi also decreased 1-MNA production, increased NAD^+^ levels, and inhibited adipocyte differentiation in vitro. With high specificity and membrane permeability, 5-amino-1MQ is emerging as a strong therapeutic candidate for cardiometabolic disorders [86]. Co-administration with NAD^+^ precursors may further enhance its efficacy [7]. Additionally, indole-3-propionic acid (IPA) has recently been shown to inhibit NNMT expression in a mouse model of heart failure with preserved ejection fraction. IPA supplementation improved NAM, NAD^+^, and SIRT3 levels, while reducing inflammation and oxidative stress, ultimately protecting against metabolic and diastolic dysfunction [78].

Beyond 1-MNA and NNMTi, several novel nicotinamide-competitive inhibitors—JBSNF-000028, JBSNF-000265, and JBSNF-000088—have been developed. These compounds reduce 1-MNA levels in mice [23,75,87], and high 1-MNA concentrations have been linked to type 2 diabetes mellitus and obesity in humans [88]. Administration of JBSNF-000028 and JBSNF-000088 decreased body weight, improved glucose regulation, and enhanced insulin sensitivity, thereby lowering CVD risk [23,75].

Other inhibitors include dual-substrate compounds (LL320 [89], GYZ-78 [90], MS2756 [91], CC-410 [92], AK-12 [75], MS2734 [91]) that occupy both the nicotinamide- and SAM-binding sites of NNMT, showing potent inhibition in vitro. However, their in vivo efficacy remains limited and requires further validation. More recently, macrocyclic peptides have emerged as the first allosteric NNMT inhibitors. These compounds bind non-competitively to an allosteric site, suppress NNMT activity, and downregulate 1-MNA production in cellular assays [93] (Table 2, Figure 2).

## 7. Future Directions

As outlined in this review, NNMT acts as a key metabolic regulator, and its role in CVD progression highlights its therapeutic potential in cardiometabolic disorders. Despite substantial progress, important knowledge gaps remain regarding the molecular mechanisms by which NNMT contributes to disease pathogenesis. A central challenge is resolving the dual nature of NNMT. While its metabolite 1-MNA exerts vasoprotective effects, NNMT activity simultaneously depletes NAD^+^ and elevates Hcy, both of which promote cardiovascular pathology. Future studies should therefore clarify how these opposing actions are balanced under physiological and pathological conditions. Another priority is to investigate the tissue-specific roles of NNMT, particularly in the vascular endothelium, cardiomyocytes, and metabolically active organs such as the liver and adipose tissue. Such studies will help define how NNMT contributes to distinct cardiovascular phenotypes.

On the therapeutic front, development should focus on optimizing selective NNMT inhibitors (e.g., 5-amino-1MQ and JBSNF compounds) and evaluating their combined use with NAD^+^ precursors such as NMN. This dual approach may help counterbalance the metabolic consequences of NNMT overactivity. Finally, although preclinical studies are encouraging, no clinical trials have yet targeted NNMT in CVD patients. Translation to clinical application will require well-designed trials and the identification of reliable biomarkers to monitor disease progression and therapeutic response. Addressing these challenges will be essential to realize the potential of NNMT-targeted therapies in cardiovascular medicine.

## 8. Conclusions

NNMT has emerged as a central regulator of cardiovascular health by linking NAD^+^ metabolism, epigenetic programming, and oxidative stress. Its overexpression disrupts multiple pathways: it depletes NAD^+^, impairing SIRT activity and mitochondrial function; elevates Hcy, driving oxidative stress and inflammation; and consumes methyl donors, altering the epigenetic landscape. Together, these mechanisms contribute to cardiometabolic disorders, including hyperlipidemia, hypertension, atherosclerosis, and myocardial ischemia. Preclinical studies consistently demonstrate that NNMT inhibition—through RNAi or small molecules—restores NAD^+^ levels, improves insulin sensitivity, reduces lipid accumulation, suppresses pro-inflammatory signaling, and ameliorates cardiac dysfunction. However, clinical translation remains limited. Current NNMT inhibitors face challenges such as poor pharmacokinetics and insufficient validation in human studies. This underscores the need for innovative drug development and carefully designed clinical trials. In summary, NNMT represents a promising therapeutic target in CVD. Future research should clarify its dual effects, optimize selective inhibitors, and advance to clinical testing. Such efforts may ultimately yield novel therapies that restore metabolic balance and improve cardiovascular health.

## Figures and Tables

**Table 1:** Pathogenic roles and regulatory mechanisms of NNMT in cardiovascular and metabolic diseases.

Disease/Condition	Pathogenic Role of NNMT	Key Mechanisms Influenced by NNMT	Regulatory Effects
**Hyperlipidemia**	Dysregulated lipid metabolism [49]; fat accumulation [16,50]	Fat metabolism [18]; resting energy expenditure [12]; elevated plasma Hcy [49]; de novo lipogenesis [50]	NNMT knockdown downregulates *Srebf1* [48] and suppresses transcriptional activity of *Lpl*, *Slc27a1*, *Fasn*, and *Fabp4* [51]
**Atherosclerosis**	Plaque instability; vascular inflammation [43]; endothelial dysfunction [21]; oxidative stress [15,19]	Hcy metabolism [52]; STAT3–IL-1β–PGE_2_ pathway [43]; NNMT–1-MNA pathway [52]	NNMT upregulation increases expression of COX-2 and PGE_2_ [43]
**Hypertension**	Endothelial dysfunction [21]; HHcy [53]	NNMT–1-MNA pathway [39]; elevated plasma Hcy [54]; reduced NAD^+^ biosynthesis [55]	Epigenetic modulation [56]
**Myocardial Ischemia**	Mitochondrial dysfunction [57]; oxidative damage [58]; ischemia–reperfusion injury [59]	NAD^+^ metabolism [2,43,60]; NMN depletion [59]; energy-related and inflammatory pathways; elevated plasma Hcy [61]	NNMT overactivation reduces *Sirt3* expression [62]; NNMT knockdown increases *Nampt* expression [18]

COX-2—cyclooxygenase-2; *Fabp4*—fatty acid binding protein 4; *Fasn*—fatty acid synthase; Hcy—homocysteine; HHcy—hyperhomocysteinemia; IL1-β—interleukin-1 beta; *Lpl*—lipoprotein lipase; MNA—1-methylnicotinamide; NAD^+^—nicotinamide adenine dinucleotide; *Nampt*—nicotinamide phosphoribosyltransferase; NMN—nicotinamide mononucleotide; NNMT—nicotinamide N-methyltransferase; PGE_2_—prostaglandin E_2_; *Sirt3*—sirtuin 3; *Slc27a1*—solute carrier family 27 member 1; *Srebf1*—sterol regulatory element-binding transcription factor; STAT3—signal transducer and activator of transcription 3.

**Table 2:** Overview of NNMT inhibitors, their half maximal inhibitory concentration (IC_50_) values, mechanisms of action, and associated disease targets.

Category	Inhibitor	IC_50_ (μM)	Role of Inhibitor	Targeted Condition(s)
**SAM-Competitive**	SAH [6]	35.3 ± 5.5 [90]	Reduces NAM methylation; preserves NAD^+^ levels [15,60]	N/A
**NAM-Competitive**	1-MNA [18,24]	24.6 ± 3.2 [94]	Reduces inflammation, oxidative stress, vascular injury [31,95]	Obesity-related cardiac injury [31]; atherosclerosis [3,91]; cardiac fibrosis [31]; hepatic ischemia–reperfusion injury [95]
	5-amino-1MQ (NNMTi) [86,96]	1.2 ± 0.1 [6]	Improves metabolic parameters; enhances insulin sensitivity [86]	Obesity-related metabolic syndrome [86]
	JBSNF-000088 [23]	2.4 ± 0.1 [6]	Restores glucose tolerance; reduces body weight [23]	Diet-induced obesity [23]
**Dual-Substrate**	CC-410 [92]	N/A	Inhibits pre-adipocyte differentiation; modulates glucocorticoid signaling [92]	Glucocorticoid-induced obesity [92]
**Allosteric**	Macrocyclic peptides [93]	0.229 ± 0.007 [6]	Non-competitive inhibition; downregulates 1-MNA production [93]	N/A

5-amino-1MQ—5-amino-1-methylquinolinium; IC_50_—half maximal inhibitory concentration; 1-MNA—1-methylnicotinamide; N/A—not applicable; NAD^+^—nicotinamide adenine dinucleotide; NAM—nicotinamide; NNMTi—nicotinamide N-methyltransferase inhibitor; SAH—S-adenosyl-homocysteine; SAM—S-adenosylmethionine.

## Data Availability

Not applicable.
